# Tumeurs parotidiennes négligées: rapport de cas

**DOI:** 10.11604/pamj.2022.43.184.29628

**Published:** 2022-12-08

**Authors:** Patrick Maholisoa Randrianandraina, Georges Franck Angelo Razanakoto, Herimalalaniaina Angelo Valisoa, Herisitraka Raotoson, Hery Henintsoa Randrianirina, Fanomezantsoa Andriamparany Rakoto

**Affiliations:** 1 Service d’Oto-Rhino-Laryngologie et Chirurgie Cervico-Faciale, Centre Hospitalier Universitaire Professeur Zafisaona Gabriel Androva, Mahajanga, Madagascar,; 2 Service d´Oto-Rhino-Laryngologie et Chirurgie Cervico-Faciale, Centre Hospitalier de Soavinandriana, Antananarivo, Madagascar,; 3 Service d´Oto-Rhino-Laryngologie et Chirurgie Cervico-Faciale Centre Hospitalier Universitaire d´Anosiala, Antananarivo, Madagascar,; 4 Service de Stomatologie et Chirurgie Maxillo-faciale, Centre Hospitalier Universitaire Professeur Zafisaona Gabriel Androva, Mahajanga, Madagascar,; 5 Service d´Anesthésie-Réanimation, Centre Hospitalier Universitaire Professeur Zafisaona Gabriel Androva, Mahajanga, Madagascar

**Keywords:** Adénome pléomorphe, tumeurs salivaires, tumeurs parotidiennes, cas clinique, Pleomorphic adenoma, salivary tumors, parotid tumors, case report

## Abstract

Les tumeurs de la glande parotide peuvent se compliquer en absence de traitement. Nous rapportons trois premières présentations cliniques et thérapeutiques malgaches de tumeurs parotidiennes négligées. Ces cas survenaient chez deux femmes et un homme de 42, 47 et 51 ans, présentant des tumeurs évoluant respectivement depuis 4, 7 et 11 ans. Une tumeur maligne était suspectée chez un patient devant la mise en évidence de nécroses cutanées, confirmée par la cytoponction. Sur consentement des patients, une parotidectomie totale était effectuée pour chaque cas. Un sacrifice du nerf facial avec évidement cervical ganglionnaire était décidé chez le patient présentant une tumeur maligne. Aucun incident peropératoire n´était noté. Les tumeurs parotidiennes peuvent se transformer en cancer en absence de traitement adéquat. Des facteurs socio-économiques sont impliqués dans le retard de consultation. Cependant, une consultation précoce devant toute tumeur parotidienne permettrait d´éviter les aléas de la prise en charge.

## Abstract (en)

Tumors of the parotid gland can be challenging if left untreated. We report three cases of patients with initial clinical and therapeutic manifestations of neglected parotid tumours in Madagascar: two women and one man aged 42, 47 and 51 years, with tumours that had been developing for 4, 7 and 11 years respectively. One patient was suspected to have malignant tumour due to skin necrosis. The diagnosis was then confirmed by cytopuncture. After obtaining the informed consent, total parotidectomy was performed in all patients. Facial nerve sacrifice with cervical lymph node dissection was established in the patient with a malignant tumour. No intraoperative adverse events were reported. Parotid tumors can become cancerous without proper treatment. Socio-economic factors are involved in delayed consultation. However, early consultation in patients with parotid tumors would prevent management risks.

## Introduction

Les tumeurs des glandes salivaires sont rares et représentent moins de 3% des tumeurs cervico faciales [1]. La glande parotide constitue le siège le plus fréquent de ces tumeurs dans 70 à 80% des cas [1,2]. Ces tumeurs d´évolution lente, sont le plus souvent bénignes, indolores et n´entrainent que rarement des gènes fonctionnels. Non traitées, elles peuvent devenir géantes et être responsables d´un gêne esthétique ou même se transformer en une tumeur maligne [3]. A travers la présente étude, nous rapportons trois cas de tumeurs parotidiennes négligées à travers lesquelles nous mettons en évidence les aspects cliniques et thérapeutiques associés à ce retard de prise en charge dans un centre de santé malgache.

## Patient et observation

### Observation 1

**Informations relatives au patient:** une femme de 51 ans s´est présentée au service d´ORL et de chirurgie cervico faciale du CHU Professeur Zafisona Gabriel de Mahajanga pour une masse latéro-cervicale gauche évoluant depuis 11 ans. Cette vendeuse ambulante, sans antécédent particulier, consultait car, gênée par le poids de la tumeur, elle ne pouvait plus porter ses marchandises sur la tête. Aucun traitement n´a été entrepris depuis selon la patiente, à part une notion de massage régulière de la tumeur.

**Figure 1:**
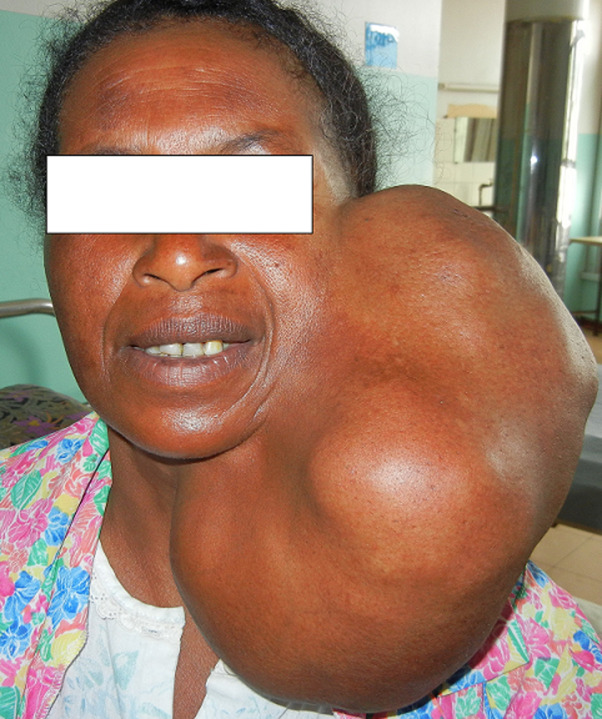
volumineuse tumeur parotidienne homogène gauche (observation 1)

**Résultats cliniques:** à l´examen, elle était en bon état général, et présentait une tuméfaction homogène, ferme de dimension 26cm x 21cm x 17cm, pendante à la partie latérocervicale gauche (Figure 1). La peau en regard était normale, il n´y avait ni de paralysie faciale, ni de dyspnée, ni gêne à la déglutition, ni d´adénopathie cervicale. L´examen endobuccal et oropharyngé étaient normaux.

**Démarche diagnostique:** le scanner cervico facial n´existait pas sur place, l´échographie cervicale rapportait une tumeur mixte aux dépens de la glande parotide gauche.

**Intervention thérapeutique:** une parotidectomie totale avec conservation du nerf facial était effectuée sur consentement de la patiente. L´hémostase était assurée par la dissection et la ligature des pédicules vasculaires tumorales, permettant une stabilité des paramètres vitaux pendant l´intervention. A l´issue de l´extraction, la tumeur pesait 9,7kg et présentait une apparence jaunâtre avec des micro calcifications.

**Suivi et résultats:** les suites étaient simples, malgré une parésie faciale temporaire de grade II de Housse et Brackmann. La patiente a pu sortir au 5^e^ jour post-opératoire. L´examen histologique de la pièce opératoire a confirmé un adénome pléomorphe sans signe de malignité. Aucune récidive n´est notée à 1 an post-opératoire.

### Observation 2

**Informations relatives au patient:** deux mois plus tard, une femme de 47 ans s´est présentée dans le même service pour une masse latéro faciale droite évoluant depuis 7 ans. Cette patiente n´avait aucun antécédent particulier, et n´avait encore reçu aucun traitement pour cette tumeur.

**Figure 2:**
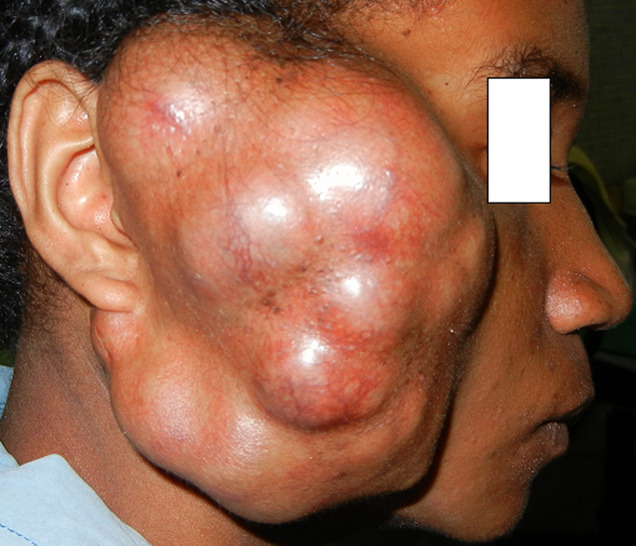
volumineuse tumeur parotidienne polylobée droite (observation 2)

**Résultats cliniques:** elle était en bon état général et présentait à l´examen une tuméfaction parotidienne droite ferme, polylobée, recouverte d´une peau saine, mesurant 15cm x 11cm x 12cm (Figure 2). L´examen endobuccal et l’examen oropharyngé étaient normaux, il n´y avait pas de paralysie faciale ni d´adénopathie cervicale.

**Démarche diagnostic:** aucun bilan d´imagerie n´avait été effectué, faute de moyens financiers et d´absence de scanner.

**Intervention thérapeutique:** une exérèse chirurgicale était effectuée avec consentement de la patiente. A l´issue de cette parotidectomie totale avec dissection et préservation du nerf facial, et malgré l´absence d´examen extemporané, la tumeur pesait 1,9kg.

**Suivi et résultats:** les suites opératoires immédiates étaient simples, il n´y avait pas de paralysie post opératoire ni d´hématome du site opératoire. La patiente a pu sortir au 5^e^ jour post-opératoire. L´examen histologique de la pièce opératoire a rapporté un adénome pléomorphe de la glande parotide sans signe de malignité. Aucune récidive n´est retrouvée à 6 mois post opératoire.

### Observation 3

**Informations relatives au patient:** il s´agissait d´un homme de 42 ans qui s´est présenté en consultation, 6 mois après le premier cas, pour une masse latéro cervicale gauche évoluant depuis 4 ans. A l´issue de séances de massages locaux, cette tumeur, indolore au départ, avait augmenté de volume avec apparition de fistules, de nécroses cutanées et de douleurs tumorales intermittentes, 9 mois auparavant, ayant motivé cette consultation. Ce patient n´avait aucun antécédent familial ni personnel particulier.

**Figure 3:**
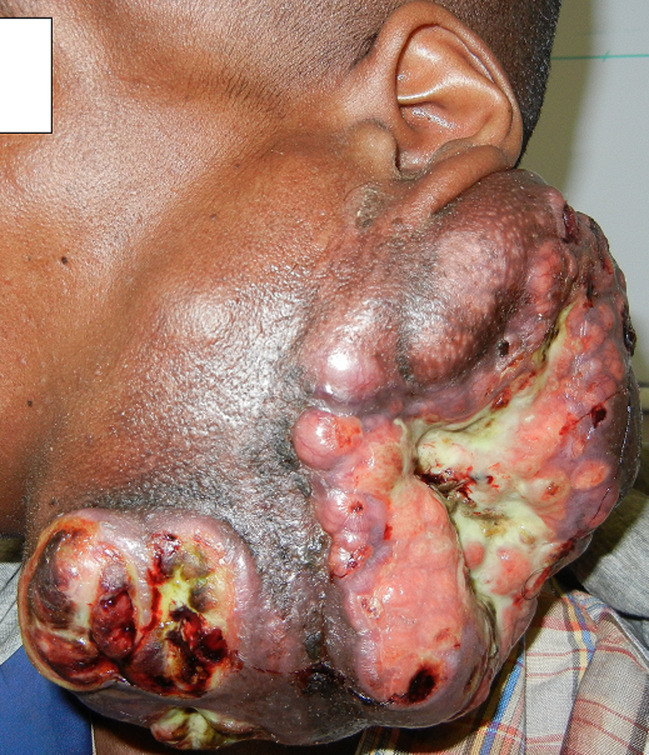
tumeur parotidienne gauche ulcéro-bourgeonnante avec nécrose cutanée (observation 3)

**Démarche diagnostic:** à l´examen, le patient était en assez bon état général, il présentait une masse parotidienne gauche, mesurant 21cm x 12,5cm x 9cm, ferme, nécrosée et bourgeonnante, saignant facilement au contact. Les foyers de nécroses étaient multiples entrecoupés d´ilots de peau saine (Figure 3). L´examen endobuccal et l’examen oropharyngé étaient normaux. Il n´y avait pas de paralysie faciale ni de gêne à la déglutition et à la respiration, il n´y avait pas d´adénopathie cervicale palpée. En absence de scanner et d'*imagerie* *par* *résonance* *magnétique* (IRM) sur place, la radiographie de la face ne retrouvait pas d´effraction osseuse. L´échographie cervicale confirmait l´origine parotidienne de la tumeur qui était hypoéchogène sans lésion kystique. Les gros vaisseaux étaient perméables. La cytoponction à l´aiguille fine suspectait une tumeur maligne de la parotide. Il n´y avait pas de signes de métastases viscérales. La tumeur était alors classée T4aN0M0.

**Intervention thérapeutique:** après consentement du patient et information sur la probable paralysie faciale post-opératoire, une parotidectomie totale non conservatrice avec évidement ganglionnaire cervicale avait été effectuée en un temps. La tumeur pesait 3,2Kg et l´examen histologique extemporané étant indisponible sur place, l´exérèse cutanée respectait une marge de 2cm en zone saine emportant la tumeur avec reconstruction par lambeaux locaux.

**Suivi et résultats:** une paralysie faciale totale était effective en période post opératoire, le patient a pu sortir au 7^e^ jour post-opératoire. L´examen histologique a confirmé un carcinome invasif au sein d´un adénome pléomorphe. Malgré une radiothérapie prévue, le patient n´avait pas pu en bénéficier. Une bonne cicatrisation cutanée ainsi qu´une absence de récidive étaient notées à 6 mois post opératoire.

**Perspective des patients:** à l´issue du traitement hospitalier, les patients ont été satisfaits car ils pouvaient reprendre leurs activités quotidiennes et une vie sociale normale, étant débarrassés des énormes tumeurs.

**Consentement éclairé:** les patients ont été informés des intérêts du rapport de leurs cas et de la particularité de la présentation de leurs pathologies. Ils ont volontairement donné leurs consentements pour permettre aux auteurs d´utiliser leurs photos pour ce rapport de cas, en respectant l´anonymat. Des consentements éclairés ont été obtenus de chaque patient pour l´utilisation de leurs photos.

## Discussion

Les tumeurs parotidiennes géantes sont rares et sont le plus souvent rapportées sous formes de cas uniques [4]. Les séries de cas rapportées varient de 2 à 3 cas [5] à 15 cas [6]. Nous rapportons une première série Malgache de tumeurs géantes de la glande parotide. L´étiologie exacte de ces tumeurs parotidiennes est imprécise bien que certains auteurs aient rapporté une recrudescence tumorale après 15 à 20 ans d´irradiation ou en association à une infection virale [6]. La durée d´évolution des tumeurs négligées de la glande parotide avant la prise en charge hospitalière varierait de 5 à 20 ans [7] s´il était de 4 à 11 ans chez nos cas. Ce retard de consultation serait lié au bas niveau socio-économique dans les pays en développement comme Madagascar, dans lesquels les couvertures sociales sont inexistantes, mais aussi aux difficultés d´accès aux soins chez des patients vivants dans des villages reculés, loin des centres de santé de références. Ces patients ne se déplaceraient pas dans des grandes villes devant cette tumeur qui est indolore, n´entrainant aucun gène fonctionnel [4]. Cependant, les tumeurs parotidiennes augmentent progressivement de volume en absence de prise en charge. Le seul gène provoqué est esthétique, constituant les derniers soucis des patients vivants en zone rurale. Ils ne viennent consulter que lorsque la tumeur devient gênante pour les activités quotidiennes ou lorsqu´une douleur apparait. Le retard de consultation pourrait aussi s´expliquer par une pratique courante dans les pays africains vis-à-vis des tumeurs de la face et du cou. Ces tumeurs y sont considérées comme étant des œuvres de sorcelleries, si bien que les patients optent pour l´automédication et la consultation chez le tradithérapeute [8] avant la consultation médicale.

La glande parotide, pesant normalement de 0,015 à 0,021Kg avec une dimension de 5,8 x 3,4cm peut être le siège de tumeurs pouvant mesurer jusqu´à 35cm x 28cm et peser jusqu´à 7,3kg [9]. Les dimensions tumorales augmentent avec leur durée d´évolution [1,2,7]. Le massage cervical, de pratique courante à Madagascar, provoque des microtraumatismes sur la tumeur, pouvant être responsable d´une augmentation plus rapide du volume de la tumeur, voire de transformation maligne. Cette pratique a été retrouvée chez nos patients. Cependant cette hypothèse reste à vérifier. La connaissance du diagnostic de nature en pré-opératoire est indispensable pour la décision thérapeutique. Une tumeur maligne bénéficiera d´une parotidectomie totale sacrifiant le nerf facial avec évidement cervical ganglionnaire alors que si la tumeur est bénigne, une parotidectomie superficielle serait suffisante [2]. Ce diagnostic pré-opératoire est basé sur la clinique et l´IRM couplées à la cytoponction donnant une sensibilité de 100% et une spécificité de 88% [2]. L´absence d´IRM, d´examen histologique extemporané ainsi que le niveau socio-économique bas étaient des obstacles à l´accessibilité de ces examens chez nos cas, rendant la prise en charge délicate.

L´anatomopathologie des tumeurs parotidiennes géantes est représentée par l´adénome pléomorphe dans 60 à 70% des cas. Les tumeurs malignes de la parotide sont rares et représenteraient moins de 10% des cas [2,7]. D´autre part, les adénomes pléomorphes peuvent se transformer en carcinomes dans 3 à 6% des cas [3]. Ce risque varie avec le temps d´évolution tumorale et atteint 9,5% pour une évolution de plus de 15 ans [2]. Ces tumeurs malignes représentent une hantise devant les tumeurs parotidiennes. Leur présentation clinique sous forme de masse ferme au sein de la glande parotide, diffère de peu de la présentation clinique d´un adénome pléomorphe. Le patient de l´observation 3 avait présenté une forme atypique de carcinome indifférencié de la parotide évolué, avec extension cutanée. Une perte d´expression du gène de l´adénome pléomorphe contrastant avec une surexpression du facteur de croissance épidermique serait responsable de la transformation des adénomes pléomorphes en tumeurs malignes [10]. Des études génétiques sont nécessaires pour confirmer cette hypothèse.

Le traitement des tumeurs parotidiennes géantes repose sur l´exérèse chirurgicale. Ce sont fréquemment des tumeurs hyper vascularisées dont l´exérèse est associée à une hémorragie massive peropératoire [4]. Une embolisation du pédicule vasculaire est préconisée en pré-opératoire afin de limiter les pertes sanguines [4,5,9]. Dans nos observations, malgré l´absence de l´embolisation pré-opératoire, une dissection méticuleuse associée à une ligature du pédicule vasculaire a permis d´avoir un contrôle de l´hémostase. Devant l´insuffisance de l´exploration pré-opératoire, afin d´éviter tout risque de récidive et de reprise chirurgicale dans une loge parotidienne traversée par le nerf facial, une parotidectomie totale conservatrice était décidée d´emblée sauf en cas de tumeur maligne évidente. La reconstruction cervicale en cas de tumeur négligée de la parotide est facilitée par la présence d´un excès de peau distendue par la tumeur, permettant la réalisation de lambeaux locaux. Une radiothérapie post opératoire est indiquée en cas de tumeur maligne invasive de la glande parotide, nécessitant ainsi un suivi au long cours. Le soulagement et la disparition de la tumeur en post opératoire associés à un contexte socioéconomique bas ont contribués aux difficultés de réalisation des traitements complémentaires post opératoires.

## Conclusion

Les tumeurs parotidiennes peuvent être très volumineuses ou se transformer en cancer en absence de traitement chirurgical. Des facteurs socio-économiques sont impliqués dans le retard de consultation. Malgré un plateau technique insuffisant, la chirurgie des tumeurs parotidiennes négligées, parfois compliquée d´hémorragie ou de blessure du nerf facial en peropératoire, peut être réalisée en respectant méticuleusement les temps opératoires. Cependant, une consultation précoce devant toute tumeur parotidienne permettrait d´éviter ces aléas de la prise en charge.
